# Increased potentiation of 5-fluorouracil induced thymidylate synthase inhibition by 5,10-methylenetetrahydrofolate (arfolitixorin) compared to leucovorin in patients with colorectal liver metastases; The Modelle-001 Trial

**DOI:** 10.1038/s44276-024-00111-4

**Published:** 2024-11-20

**Authors:** Helena Taflin, Elisabeth Odin, Göran Carlsson, Bengt Gustavsson, Oskar Hemmingsson, Yvonne Wettergren, Krzysztof Urbanowicz, Jacek Turyn, Ryszard T. Smolenski, Godefridus J. Peters

**Affiliations:** 1 Department of Surgery, Institute of Clinical Sciences, Sahlgrenska Academy, University of Gothenburg, Gothenburg, Sweden; 2 Region Västra Götaland, Sahlgrenska University Hospital, Department of Surgery, Gothenburg, Sweden; 3 Department of Diagnostics and Intervention/Surgery, Umeå University, Umeå, Sweden; 4 Department of Biochemistry, Medical University of Gdansk, Gdansk, Poland; 5 Department of Medical Oncology, Cancer Center Amsterdam, Amsterdam University Medical Centers (Amsterdam UMC), Vrije Universiteit Amsterdam, Amsterdam, the Netherlands

## Abstract

**Background:**

5-Fluorouracil (5-FU) is a cornerstone in treatment of colorectal cancer (CRC) and is usually combined with leucovorin (LV) to enhance the antitumour effect by increase thymidylate synthase (TS) inhibition, the key target enzyme for 5-FU. Arfolitixorin (Arfo) is an active form of the reduced folate, [6 R]-5,10-methylenetetrahydrofolate ([6 R]-MeTHF and in contrast to LV, does not need to be metabolized. The Modelle-001 was designed to explore whether a single intravenous bolus injection of Arfo as compared to LV, together with 5-FU increases the inhibition of TS, levels of folate concentrations and polyglutamylation in CRC liver metastases (CRLM) and liver parenchyma.

**Patients and methods:**

Thirty patients with CRLM received either LV (60 mg/m^2^) or Arfo (30 mg/m^2^ or 120 mg/m^2^) in combination with 5-FU preoperatively. Levels of folates and and TS inhibition were measured.

**Results:**

Significantly higher MeTHF levels and higher TS inhibition were measured in the Arfo groups compared to LV60, and there was a difference in folate poly-glutamylation between the groups.

**Conclusion:**

The Modelle-001 Trial demonstrated significantly higher levels of MeTHF in metastases following Arfo compared to LV. This resulted in a greater increase TS inhibition in metastases although not statistically significant.

## Introduction

Colorectal cancer (CRC) is the third most common cancer worldwide with an incidence of 1.1 million new cases per year and is the second leading cause of cancer-related deaths [1]. Chemotherapy is used in all settings; neoadjuvant to reduce the tumor burden prior to surgery, adjuvant after radical surgery to reduce the risk of recurrence, and in the palliative setting with the purpose of prolonging life. Chemotherapy is also commonly used before surgical treatment of colorectal liver metastases (CRLM), to stabilize or downsize the metastatic burden [2].

Several novel combination treatments have been developed during the last decades, e.g., 5-fluorouracil (5-FU) and the reduced folate leucovorin (LV) in combination with oxaliplatin (FOLFOX) or irinotecan (FOLFIRI). Antibodies directed against the epidermal growth factor receptor may be added for tumors with wild-type KRAS and BRAF genotype. However, 5-FU is still the basis for treatment of CRLM as well as several other gastrointestinal cancers [3]. The effect of 5-FU is increased (response rate and overall survival) by concurrent (or pre)-treatment with (LV) [4, 5]. A racemic mixture of the natural (S) and unnatural (R) diastereoisomers of LV is most often administered, usually in the form of calcium folinate. LV is metabolized intracellularly to the active metabolite 5,10-methylenetetrahydrofolate (MeTHF). MeTHF is the methyl-donor essential for the action of thymidylate synthase (TS), which catalyzes the reductive methylation of deoxyuridine monophosphate (dUMP) to deoxythymidine monophosphate (dTMP). dTMP is subsequently phosphorylated to deoxythymidine triphosphate (dTTP), which is required for DNA replication and repair [6–8].

After entering the cells, 5-FU is converted to 5-fluoro-2′-deoxyuridine 5′-monophosphate (FdUMP), which forms a stable ternary complex between TS, FdUMP, and MeTHF leading to inhibition of the TS enzyme activity. Since the rate-limiting TS inhibition exploits one of the few metabolic bottlenecks in DNA synthesis, drugs inhibiting TS can be considered as first examples of targeted therapy [6, 9].

The FdUMP-TS complex formation and stability are dependent upon the cellular concentration of both MeTHF and FdUMP. In addition, the effect of MeTHF increases by the level of its polyglutamylation, by attachment of up to five glutamate molecules [10, 11]. A combined regimen of 5-FU and LV leads to an increased intracellular pool of MeTHF and enhanced 5-FU cytotoxicity, as this favors the equilibrium towards FdUMP-TS ternary complex formation, ensuring maximal TS inhibition. Potentiation of 5-FU-induced TS inhibition by LV has been found in many model systems, including cell lines and animal model systems for CRC, as well as in CRC patients [12–16].

However, the LV-mediated increase of TS inhibition is limited by its tissue distribution and subsequent cellular uptake and metabolism to MeTHF. It has been hypothesized that direct administration of MeTHF would bypass the limiting steps in metabolism. Arfolitixorin ([6 R]5,10-methylenetetrahydrofolate) (Arfo) was designed to replace reduced folates such as LV as biomodulator. The compound is a hemisulfate salt of MeTHF that makes the folate more stable compared to other salts. In contrast to LV, Arfo does not require enzymatic metabolic activation because it constitutes the active folate of the ternary complex. However, Arfo still requires cellular uptake mediated by the reduced folate carrier (RFC), which is high in most tissues, including colon tumors and colon cancer cell lines [16, 17]. Arfo can be trapped in these tissues by polyglutamylation, mediated by folylpolyglutamate synthetase (FPGS) which is variable but usually high in these tissues as well [8, 16, 18]. Patients who are incapable of metabolizing LV sufficiently might thus benefit from Arfo administration.

The Modelle-001 study was designed as a proof-of-principle study to explore whether a single intravenous bolus injection of Arfo as compared to LV, together with 5-FU would increase the inhibition of TS in CRLM and adjacent liver parenchyma. The study further aimed to explore whether administration of Arfo would lead to a higher accumulation of MeTHF in the metastases compared to LV and whether this would affect folate concentration and polyglutamylation in tissues.

## Patients and Methods

### Patients

Thirty adult patients with biopsy-verified colorectal cancer and a minimum of two liver metastases, indicated for surgical removal were enrolled at two Swedish University Hospitals: Sahlgrenska University Hospital and Norrland University Hospital. Both synchronous and metachronous liver metastases were accepted and the majority of patients had been subjected to preoperative chemotherapy, although with a pause of at least four weeks prior to surgery according to clinical routine. One additional patient received study folate but was excluded from peroperative chemotherapy due to massive tumor burden and need of extended liver surgery. This patient was replaced according to the intention to treat plan and 30 patients met the final inclusion criteria.

The study was conducted in three phases as illustrated in Supplementary Fig. 1. The initial six patients received either 60 mg/m^2^ LV or 30 mg/m^2^ Arfo at a median time of 87 min (range 81–107) before administration of a reduced dose of 250 mg/m² 5-FU in order to evaluate safety, since peroperative chemotherapy is not a standard procedure for resection of CRLM. However, in an earlier study, which combined a bolus of 5-FU at 500 mg/m^2^ given in the middle of a 2 h infusion with racemic LV at 500 mg/m^2^, no adverse events were found [12]. When all six patients had been followed-up in the context of visit consultations, a safety evaluation of all adverse events/serious adverse events was performed by an independent liver surgeon and an oncologist. The six patients who received 250 mg/m^2^ 5-FU were not included in tissue analyses. As the study was allowed to continue, the next 24 patients received 60 mg/m^2^ LV, 30 mg/m^2^ Arfo or 120 mg/m^2^ Arfo at a median time of 82 min (range 19–300) before 500 mg/m² 5-FU, with a maximal dose of 1 g, which is in accordance to the Nordic bolus regimen [19]. Start and stop times of both folate and 5-FU administration were recorded.

At the day of the surgery, the patients were randomized by sealed envelope to receive either LV or Arfo by a special team of research nurses outside the department to keep the study blinded. The folates were administered in a central venous line as a short bolus injection (2–3 min) and blood samples were drawn from an arterial catheter in chilled S-Monovette K3 EDTA tubes containing 0.1% ascorbate before (time 0) and 10, 20, 30, and 60 min after intravenous bolus injection of folates. The tubes were inverted gently 10 times, then put on wet ice, protected from light. Plasma was isolated from blood by centrifugation within 30 min at 1520 g for 10 min, 4 °C. The plasma samples were immediately frozen and stored at −80 °C until used.

Biopsies from the first metastasis and adjacent liver parenchyma were collected after a minimum of 60 min past injection of either LV or Arfo. Thereafter, an individualized 5-FU bolus injection based on body surface area was administered in a central venous line. To prevent exposure of 5-FU-contaminated blood to the operative team, at least 60 min had to elapse between the 5-FU injection and resection of the second metastasis and adjacent liver parenchyma. This pause was also important to ensure formation of FdUMP and to form the ternary complex (FdUMP-MeTHF-TS). Earlier studies showed that 5-FU would be taken up rapidly in liver metastases immediately leading to the formation of FdUMP, enabling its binding to the ternary complex [13, 20]. Tissues were snap-frozen on dry ice directly in the operating room and kept at −80 °C until analyzed.

### Stability test of Arfo in blood samples by adding ascorbate

A comparative test was performed to analyze the stability of Arfo in blood by either adding 0.1% ascorbate alone (test A) or 555 µl (0.1%) ascorbate plus 112 µl 2,3-dimercapto-1-propanol (test B) to 5 ml blood. Three concentrations (100, 10 and 1 µM) of Arfo were added to the samples and the MeTHF levels were analyzed on LC-MS/MS (see below) after 0, 10, 20, 30, and 60 min. After 60 min, the variation [(SD/mean)x100] of MeTHF was found to be 15, 18 and 16%, respectively, in test A, and 5, 7 and 9%, respectively, in test B. However, hemolysis caused by the 2,3-dimercapto-1-propanol was noted in all blood samples of test B. Due to the cell lysis, it was concluded that the measured folate concentrations do not reflect the actual plasma folate levels. Thus, for practical reasons (the odor and toxicity of the substance was not desirable at the operation room), and despite the higher variability, we chose to add 0.1% ascorbate alone to the blood samples of the present study.

### Plasma folate analyses

Folate analyses plasma was performed as described previously [21, 22] with minor modifications.

Ten µl of distilled water, 10 µl of the internal standard (IS) aminoacetophenone, and 250 µl of 10 mM TCEP in Methanol:DMSO (50:50) were added to 50 µl plasma. The samples were mixed and centrifuged at 21,500 g, 4 °C, for 10 min. The supernatants were then analyzed by liquid chromatography electrospray ionisation tandem mass spectrometry (LC-MS/MS) as described previously [21, 22]. The AUC values (expressed as nmol/ml/h) of MeTHF, 5-formyltetrahydrofolate (FTHF), methyltetrahydrofolate (MTHF), and tetrahydrofolate (THF) were calculated by measuring plasma folate concentrations at time 0 to 1 h. Intra-batch variability was determined by analyzing tissue Q-samples at low, medium, and high concentrations on the same day. Inter-assay variability was determined by analyzing low, medium, and high concentration samples on five separate days. The relative standard deviation (RSD) ranged from 1–16%, and the variability over 5 days ranged from 8 to 21%.

### Folate levels in tissue

On the day of sample analysis, extraction buffer was prepared containing 50 mM phosphate buffer (pH 7.0), 1% ascorbate, and 0.1% β-mercaptopropanol. The tissue was weighed and placed in an Eppendorf vial and a 10× volume of extraction buffer was added. Homogenisation was performed using a TissueLyzer (two disruption steps at 25 Hz for 2.5 min). Tomudex was used as an internal standard (IS). Twenty-five µl of conjugase were added to the homogenate and mixed followed by incubation at 37 °C for 60 min. The monoglutamate folate levels were analyzed by adding 25 µl extraction buffer (instead of conjugase) to a separate part of the homogenate, followed by mixing and incubation at 37 °C for 60 min. Deconjugation, was followed by protein precipitation, centrifugation and ultrafiltration (30 min at 21,500 g, 20 °C). The solution at the bottom of the test tube was used for the LC–MS/MS analysis. The difference between the samples treated with conjugase and the untreated samples was designated as polyglutamylated folates and these levels were used to calculate the ratio poly-/monoglutamates.

Calibration was performed by plotting the concentration against the peak area ratio of each compound to internal standards. The standards and samples were processed using the QuanLynx quantitative processing tool in MassLynx (Waters Corp., Milford, MA, USA). Intra-batch variability was determined by analysing tissue Q-samples at low, medium, and high concentrations on the same day. Inter-assay variability was determined by analysing low, medium, and high concentration samples on 9 separate days. The relative standard deviation (RSD) ranged from 0.2 to 13.5%, and the variability over 9 days ranged from 1.6 to 12.9%. The levels of MeTHF, FTHF, MTHF and THF in each sample were expressed as nmol/g wet-weight (nmol/g w.w.).

LC-MS/MS was used to evaluate the levels of the folate derivatives (MeTHF, FTHF, MTHF, and THF) in plasma, metastatic tissue, and adjacent liver parenchyma, as described previously [21, 22]. Briefly, the LC-MS/MS analyses were performed on a 2795 LC separation module coupled to a Micromass Quattro Triple-Quadrupole MS system with an electrospray ionisation (ESI) source (Waters). Folates were detected and quantified using positive electrospray. The separation of folates was performed using a 3 µm Atlantis dC_18_, 2.1 × 100 mm column (Waters) together with the guard column Atlantis dC_18_, 3 µm, 2.1 × 10 mm. The mobile phase consisted of eluent A (0.1% acetic acid in water) and eluent B (0.1% acetic acid in acetonitrile). The extracted ions following MRM transitions were monitored at m/z 474 → 327 for FTHF, m/z 458.15 → 311 for MeTHF, m/z 460.14 → 313.1 for MTHF, m/z 446 → 299 for THF, m/z 459.06 → 312.09 for Tomudex (IS, tissue), and m/z 136 → 94.1 for isophenone (IS, plasma).

### FdUMP levels in tissue

FdUMP analysis was performed as described previously [23] with minor modifications.

A 700 µl mixture consisting of 90% methanol and 0.1% acetic acid (HAc) was added to each tissue sample. The samples were then homogenized for 2.5 min at a frequency of 25 Hz using a TissueLyzer (Qiagen), followed by centrifugation for 5 min at 3000 g. The supernatants were transferred to separate tubes and 10 µl of 1 mM CldUr (IS) were added. To each remaining pellet, a 700 µl mixture consisting of 90% methanol and 0.1% HAc was added, and the samples were again homogenized for 2.5 min at 25 Hz, followed by centrifugation for 5 min at 3000 g. The pooled supernatants were evaporated, then dissolved in 300 µl of distilled H_2_O by mixing for 10 min at 37 °C. The samples were then centrifuged for 10 min at 21,500 g, 8 °C and the supernatants were analysed by LC-MS/MS. FdUMP was detected using negative electrospray. The eluent used for FdUMP analyses was an isocratic elution consisting of 95% A: (0.1% HAc) and 5% B: (0.1% HAc in 90% acetonitrile). The extracted ions following MRM transition were monitored at m/z 325 → 195 for FdUMP and 261 → 171 for CldUr (IS).

### TS activity and TS inhibition

TS activity assays were performed according to an LC-MS-based protocol [24]. Assay condition and sample preparation are based on a previously described assay using tritiated FdUMP [12, 13, 15] and were adapted for the LC-MS/MS assay.

Tissues weighing between 50 and 80 mg were homogenized and suspended in an assay buffer consisting of 200 mM TRIS, 100 mM sodium fluoride, 15 mM cytidine monophosphate and 20 mM 2-mercaptoethanol (pH 7.4) at a ratio of 30 **µ**l buffer for 10 mg of tissue. The suspension was then centrifuged at 1500 g at 4 **°C** for 10 min. The resulting supernatant was centrifuged again at 18,000 g at 4 °C for 10 min while the pellet was stored at −80 °C.

For the assay, 7.5 µl of the assay buffer was added to 12.5 µl of the 18,000 g supernatant, followed by the addition of 5 µl of 5 µM dUMP. Protein content was measured in parallel using the Lowry method. The activity assay was performed at 37 °C for 60 min. Reactions were stopped by addition of 100 µl of pre-chilled methanol. After vortexing for 15 s samples were chilled on ice for 20 min and subsequently centrifuged for 15 min at 18,000 g at 4 °C. The collected supernatant was vacuum-dried and stored at −80 °C until analysis. On the day of the analysis the analytes were dissolved in 50 µl of 0.1% formic acid containing 1 µM 2-chloroadenosine as internal standard. After vortexing for 30 s the samples were centrifuged for 15 min at 18,000 g and 4 °C. Afterwards, 35 µl of the supernatant was collected and submitted for LC-MS analysis, as described.

### Statistical analysis

Statistical analyses were performed using the JMP Pro 17.0.0/SAS software (SAS Institute Inc. Cary, NC, USA) or the GraphPad Prism 10.0.2 software (Boston, MA, USA). Data were presented as median and ranges. Due to large interindividual differences in time passed after folate injection to biopsy collection within each group, the folate concentrations were adjusted by the covariate “time” in the statistical calculations. Furthermore, the poly-/monoglutamylated folate ratios were adjusted by the covariate “total tissue folate concentration” since the folate concentration *per se* may impact the cellular demand for folate polyglutamylation. Finally, because of the large interindividual differences in tissue FdUMP levels, which have large impact on TS inhibition, the FdUMP concentration was used as a covariate in the statistical calculation of TS inhibition. TS inhibition was calculated from TS activity using the formula *[1 – (TS activity after 5-FU/TS activity before 5-FU) X 100]*. Differences between groups were analyzed using the Anova t-test or the Wilcoxon rank sum test when using adjusted values. Pairwise comparisons were performed using the Wilcoxon matched-pairs signed rank test. Values of *p* < 0.05 were considered significant.

## Results

Patient and tumour characteristics are presented in Table 1.

**Table 1:** Patient and tumor characteristics.

Number of included patients (*n*)	30^a^
Age (years), median (range)	67 (45–81)
Male/Female (*n*)	16/14
Primary tumour location (*n*)	
*Right Colon*	2
*Left Colon/Sigmoid*	13
*Rectal*	15
Preoperative chemotherapy (*n*)	26
KRAS/BRAF mutation (*n*)	13

^a^One additional patient received study folate but was excluded from preoperative chemotherapy and not valid for inclusion.

A total of 50 adverse events (AE) were defined of which 11 were considered as serious adverse events (SAE). Most of the adverse events were mild infections or light fever, the serious adverse events had no clear pattern. None of the AE/SAEs was defined as a suspected unexpected serious adverse reaction (SUSAR) and the number of AE/SAEs was considered proportional to expected complications after surgical resection of liver metastases.

### Plasma folate levels

**Fig. 1:**
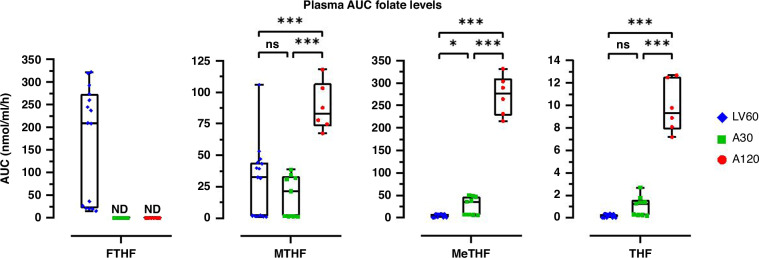
Plasma AUC folate levels after intravenous bolus injections of 60 mg/m^2^ LV (LV60, *n* = 15), 30 mg/m^2^ Arfo (A30, *n* = 9), or 120 mg/m^2^ Arfo (A120, *n* = 6)). Data are presented as box-and-whisker plots with median, 25 and 75% quantiles (box), minimum and maximum (whisker), and outliers. Symbols represents individual values. Significant differences between groups are denoted by asterisks; *p < 0.05; **p < 0.01; ***p < 0.001. ND not detected, FTHF 5-formyltetrahydrofolate, MeTHF 5,10-methylenetetrahydrofolate, MTHF 5-methyltetrahydrofolate, THF tetrahydrofolate.

The AUC levels of FTHF, MeTHF, MTHF, and THF in plasma were measured after intravenous bolus injections of LV or Arfo (Fig. 1). As expected, the median plasma AUC level of FTHF was high in the LV60 group, although one patient cluster (*n* = 6) had a level below 40 nmol/ml/h. The plasma AUC of FTHF in groups A30 and A120 were also low and below detection limit. The AUC level of MeTHF was lowest in the LV60 group, higher in the A30 group, and dose-dependently highest in the A120 group. There was no difference in AUC levels of neither MTHF nor THF between LV60 and A30, but significantly higher levels were seen in A120 compared to both groups. Two distinct clusters were seen with high and low AUC levels of MTHF (*p* = 0.0018) in LV60, where the cluster with high MTHF levels was related to the cluster with high FTHF levels. The increase in MTHF levels may have clinical relevance since MTHF and its metabolites tetrahydrofolate and dihydrofolate can also form a ternary complex with FdUMP and TS and in this way increase TS inhibition [25], which can be enhanced by their polyglutamates.

### Time until biopsy specimen collection after folate and 5-FU injections

No significant differences were seen between the groups in time passed from folate or 5-FU administration to biopsy sampling (data not shown). However, large interindividual differences were seen within each group (Supplementary Table 1).

### Total folate levels in tissues

**Fig. 2:**
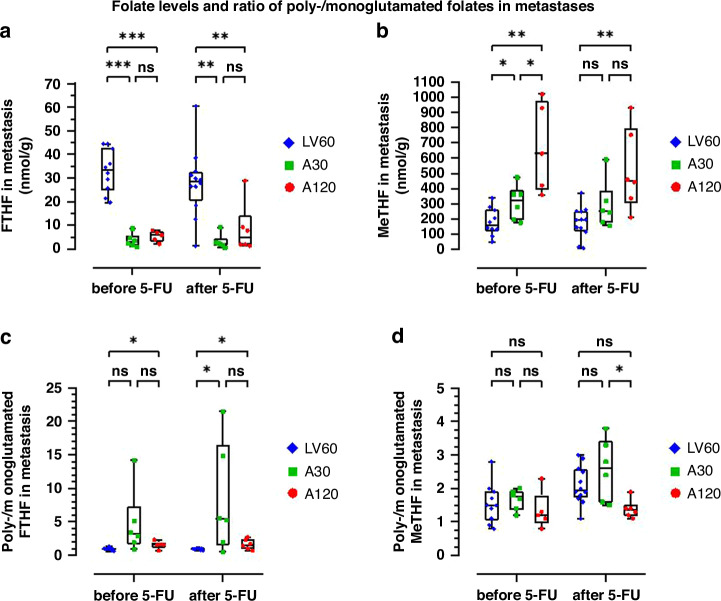
Folate levels and ration of poly/monoglutamated folates in metastases. Comparison of (**a**) total (both mono- and polyglutamylated) 5-formyltetrahydrofolate (FTHF) concentrations, (**b**) total 5,10-methylenetetrahydrofolate (MeTHF) concentrations, (**c**) the ratio poly-/monoglutamylated FTHF, and (**d**) the ratio poly-/monoglutamylated MeTHF in metastatic tissues after intravenous bolus injections of 60 mg/m^2^ LV (LV60), 30 mg/m^2^ Arfo (A30), or 120 mg/m^2^ Arfo (A120) followed by bolus injections of 500 mg/m^2^ 5-FU. Data obtained before 5-FU were lacking for two patients in LV60 and one patient in A120. Data are presented as box-and-whisker plots with median, 25 and 75% quantiles (box), minimum and maximum (whisker), and outliers. Symbols represents individual values. Significant differences between groups are denoted by asterisks; *p < 0.05; **p < 0.01; ***p < 0.001.

The concentrations of FTHF and MeTHF were determined in liver parenchyma and metastatic tissues after intravenous bolus injections of 60 mg/m^2^ LV, 30 mg/m^2^ Arfo, or 120 mg/m^2^ Arfo followed by bolus injections of 500 mg/m^2^ 5-FU. The FTHF and MeTHF concentrations were higher in liver parenchyma compared to metastases (Supplementary Fig. 2A, B and Fig. 2a, b). As expected, FTHF levels were significantly higher in metastases of patients treated with LV60 compared to groups treated with Arfo (Fig. 2a). The median level of FTHF was higher in A120 compared to A30, but the difference did not reach significance. In contrast, the MeTHF levels in metastases were significantly higher in the groups A30 and A120 compared to the LV60 group (Fig. 2b).

It is known that polyglutamylated folates are retained longer in cells compared to monoglutamylated folates and that polyglutamates of MeTHF and other (anti)folates increase the binding of FdUMP to TS [25, 26]. We therefore determined the extent of polyglutamylation by calculating the ratio poly-/monoglutamylated free FTHF and free MeTHF in liver parenchyma and metastatic tissues before and after bolus injections of 5-FU. Interestingly, it was noted that samples with the highest folate levels had the lowest mono-polyglutamylated folate ratios, both before and after 5-FU administration (Fig. 2 and Supplementary Fig. 2). However, this does not mean that the absolute polyglutamate levels were lower in the A120 group, since the mean MeTHF polyglutamate level amounted to 385 nmol/g before 5FU administration and decreased to 307 after 5-FU administration. In the A30 group these mean concentrations were 193 and 209 nmol/g, respectively, and in the LV60 group these numbers were even lower, 110 and 121 nmol/g, respectively. This indicates that in the A30 and LV60 groups polyglutamylation continued after 5-FU was given. Although in the A120 group polyglutamate levels went down, they still remained much higher compared to the A30 (1.5-fold) and LV60 (2.5-fold) groups. Also MeTHF monoglutamate levels remained much higher (222 nmol/g) in the A120 group, compared to the A30 (81 nmol/g) and LV60 groups (57 nmol/g).

The poly-/monoglutamylated FTHF ratio was significantly higher in A120 compared to LV60 before 5-FU administration in both liver parenchyma (Supplementary Fig. 2C) and metastases (Fig. 2c). After 5-FU administration, the poly-/monoglutamylated FTHF ratio was significantly higher in metastases of groups A30 and A120 compared to LV60 (Fig. 2c). However, both FTHF mono- and polyglutamate levels were much higher in the LV60 group compared to the A30 and A120 groups.

The poly-/monoglutamylated MeTHF ratio in liver parenchyma of group LV60 was significantly higher compared to group A120 before and after 5-FU administration and in group A30 compared to A120 before 5-FU (Supplementary Fig 2D). Also in metastases, the poly-/monoglutamylated MeTHF ratio was significantly higher in group A30 compared to A120 after 5-FU (Fig. 2d). The polyglutamylation of MeTHF in metastases of groups LV60 and A30 was significantly higher (*p* = 0.0039 and *p* = 0.031, respectively) after 5-FU administration as compared to before (Fig. 2d).

### FdUMP concentrations in liver parenchyma and metastatic tissue

**Fig. 3:**
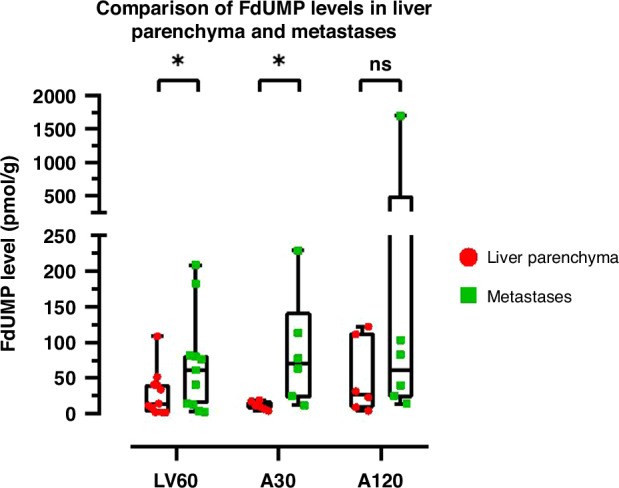
Comparison of the FdUMP concentrations in liver parenchyma and metastases after bolus injections of 500 mg/m^2^ 5-FU. Data were lacking from two patients in the LV60 group. Data are presented as box-and-whisker plots with median, 25 and 75% quantiles (box), minimum and maximum (whisker), and outliers. Symbols represents individual values.

FdUMP was rapidly formed after 5-FU administration and clearly detectable in both liver parenchyma and metastases. There was a large interindividual difference in FdUMP concentrations and, using the Wilcoxon matched-pairs signed rank test on pooled data from all groups, the levels were shown to be significantly higher in metastases compared to liver parenchyma (*p* < 0.01). The FdUMP level in treatment groups are presented in (Fig. 3). There was no significant differences in FdUMP levels between groups.

### TS activity and inhibition

The TS activities were measured in tissue samples before and after 5-FU administration enabling determination of TS inhibition in each patient. The basal levels of TS activity (before 5-FU administration) were about four times higher in metastases compared to liver parenchyma. Pairwise analysis using Wilcoxon matched-pairs signed-rank test showed no difference in TS activity measured before and after 5-FU administration in liver samples in any group.

**Fig. 4:**
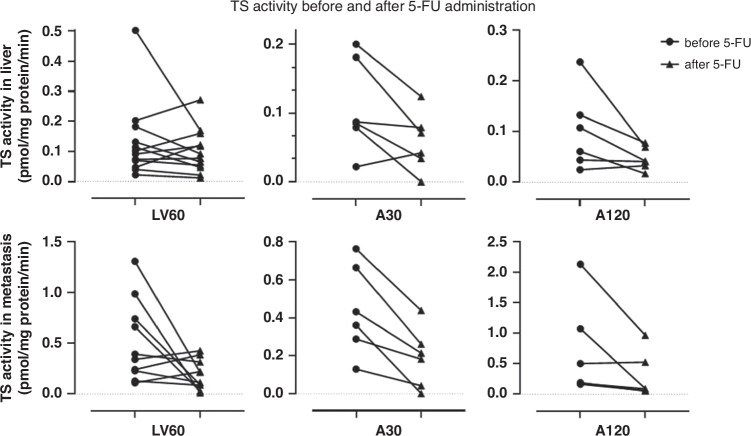
Comparison of TS activity in liver parenchyma (upper panel) and metastases (lower panel) after intravenous bolus injections of 60 mg/m^2^ LV (LV60), 30 mg/m^2^ Arfo (A30), or 120 mg/m^2^ Arfo (A120) followed by bolus injections of 500 mg/m^2^ 5-FU. Data were lacking from two patients in the LV60 group. Symbols represents individual values.

In liver parenchyma, 5-FU administration had a relatively low effect on TS activity with a minor decrease in the LV60 and A30 groups and a somewhat higher apparently dose-dependent effect in the A120 group. However, there was a significant difference in TS activity before and after 5-FU administration in metastatic tissue derived from patients of the A30 group (*p* < 0.05), but not in LV60 (*p* = 0.16) or A120 (*p* = 0.062). (Fig. 4).

When evaluated as TS inhibition (Supplementary Table 2 and Table 2), the effect of 5-FU administration was much more pronounced in liver metastases compared to liver parenchyma. Four patients had no TS inhibition in metastases and corresponding liver parenchyma (three in the LV60 group, one in the A120 group). Three additional patients had no TS inhibition in liver parenchyma (two in the LV60 group, one in the A30 group). The TS inhibition in liver parenchyma and metastases was higher in the A30 and A120 groups compared to the LV60 group however, the differences did not reach significance (Supplementary Table 2 and Table 2).

**Table 2:** TS inhibition in metastases after 5-FU injection.

Group	n	TS inhibition (%)	
		Median (range)	*p*
LV60	9	32.6 (0–95.6)	0.23^a^
A30	6	55.5 (37.0–100)	0.19^b^
A120	6	58.6 (0–91.3)	0.94^c^

^a^LV60 vs A30, ^b^A30 vs A120, ^c^LV60 vs A120.

## Discussion

In this study we have shown that Arfo, the active form of MeTHF, shows a dose dependent inhibition of TS specifically in liver metastases, which is higher than LV-mediated TS inhibition. The combination of LV and 5-FU is standard in current chemotherapy schedules [2] and response to these schedules is dependent on inhibition of the target enzyme, TS. Previous studies have shown that it is possible to modulate TS inhibition with folates [7], given either as the racemic mixture at a low and a high dose, or as pure levo-LV [27]. When given as levo-LV, the low dose of LV (25 mg/m^2^) resulted in less inhibition of TS compared to the high-dose (500 mg/m^2^).

In previous studies, we evaluated different administration methods and doses of various folate species (racemic LV, levo-LV, Arfo) (bolus or continuous infusion) [22, 28–31]. In these studies, however, folate was not given in combination with 5-FU, i.e., the possible effect on TS inhibition could not be evaluated. In one study, various folate species were evaluated in liver metastases at 48 hr after 5-FU and LV (500 mg/m^2^) administration; both MeTHF and MTHF were still 5 and 20-fold increased, respectively [27]. The present Modelle-001 trial was designed to compare the effect of two different folates (LV and Arfo) in combination with 5-FU on TS activity and inhibition in CRLM and matching liver parenchyma.

The folate doses were chosen to compare Arfo to a standard dose of LV (60 mg/m^2^) according to the Nordic bolus regimen. Since LV is a racemic substance (R and S form), whereas Arfo consists of the biologically active R form only, the Arfo dose of 30 mg/m^2^ was considered to be equivalent to LV60. The cohort receiving Arfo 120 mg/m^2^ was added to explore the effect of a higher dose. The results showed that preoperative administration of 5-FU and LV or Arfo is safe. The levels of SAE were in accordance with expected complication rates after liver resection of metastases [32]. There was no statistical difference in SAEs between the groups and there was no increase in SAE in the A120 group, which received the highest dose of Arfo.

The plasma AUC of FTHF was high in the LV60 group but below detection limit in the groups A30 and A120. The high level relates to the presence of both the unnatural (R) and natural (S) forms of FTHF. It is known that the unnatural form is retained longer in the blood [33]. The two patient clusters with high and low plasma AUC levels of FTHF identified in the LV60 group may result from different rates of FTHF metabolism in the clusters. High MTHF levels are known to inhibit the conversion of FTHF to downstream metabolites [34] and the correlation seen between high AUC values of FTHF and MTHF suggests that this might have been the case. Plasma MeTHF concentrations were lowest in the LV60 group, higher in the A30 group, and highest in the A120 group. These findings are in line with a previously conducted study, in which patients with colon cancer were randomized to receive either Arfo or Isovorin (levo-LV) [31].

As expected, the FTHF level before 5-FU was significantly higher in the LV60 group compared to A30 and A120 in both liver parenchyma and metastases, whereas the MeTHF level was highest in the A120 group. Consistent with previous studies on CRC [22, 31], these results demonstrated that there is an uptake of LV and Arfo into tissues, and that the uptake of Arfo is dose-dependent.

The poly-/monoglutamylated FTHF ratio in both liver parenchyma and metastases was low in groups LV60 and A120 compared to A30. This may be related to the lower folate concentrations seen in tissues of patients of group A30 compared to A120 group. When the folate level is high, the demand for folate polyglutamylation is low as has been shown previously in cell lines [35]. The high levels of MeTHF in liver parenchyma and metastases and low poly-/monoglutamylation ratio of MeTHF in A120 (Fig. 2b) supports this theory. There were no major differences in folate levels and polyglutamylation between the samples taken before and after 5-FU administration neither for liver parenchyma nor for metastases. The level of polyglutamylation for the A30 group seems to increase slightly over time at low folate concentrations whereas this is not the case at high folate concentrations. This result may possibly indicate that the dose of 30 mg/m^2^ Arfo was not sufficient to achieve an optimal TS inhibition.

The FdUMP concentration was significantly higher in metastases compared to liver parenchyma possibly indicating a stronger cytotoxic effect in metastases, and less side-effects in normal liver tissue. The FdUMP levels both in liver and liver metastases were comparable to those reported earlier [13, 20]. The low FdUMP level in liver is not unexpected since the level of 5-FU was also found to be much lower in liver compared to liver metastasis [20] due to high levels of the 5-FU degradation enzyme dihydropyrimidine dehydrogenase (DPD) [36–38]. The large interindividual variation in FdUMP tissue levels was observed earlier [13, 20] and can be the result of the variation in sampling times, but also be related to variations in vascularization in the liver.

The TS activity in liver metastasis as found in the Modelle-001 study were in a similar range as observed earlier with the conventional TS assay, using tritiated dUMP [12, 27]. Also the extent of TS inhibition for the combination of LV (at a high dose) and 5-FU was in the same range. Additionally, TS activity in normal liver was much lower compared to liver metastasis [12, 39] and in the same range as in the present study. Next, inhibition of TS in normal liver was minimal as was found in the present study as well.

It was hypothesized that Arfo might be superior compared to LV in the mFOLFOX regimen for metastatic CRC, which was evaluated in the AGENT study [40]. The AGENT study was a randomized, phase III study in which patients with mCRC were randomized to receive Arfo (120 mg/m^2^ given as two intravenous bolus doses of 60 mg/m^2^) or LV (400 mg/m^2^ given as a single intravenous infusion) plus 5-FU, oxaliplatin, and bevacizumab. The study did not show any difference in the primary endpoint which was overall response rate (ORR). However, Arfo was given as a split intravenous dose instead of a single high bolus as well as at a lower dose than LV. This could have led to inadequate tumor tissue levels of MeTHF, leading to an inferior TS inhibition.

A major advantage of the present study was the real-life environment, where liver surgeons and nurses enabled a standardized sampling. However, most importantly, each patient served as his own control regarding the TS measurements. The assays (both for folates and TS) were performed according to well-established methods. The present study, despite including a low number of patients, showed that administration of 120 mg/m^2^ Arfo led to higher MeTHF levels compared to 30 mg/m^2^ Arfo and 60 mg/m^2^ LV, and a higher TS inhibition in the A120 group compared to LV60 although the difference did not reach statistical significance. However, in the LV60 group in several patients no TS inhibition was observed, while in the Arfo groups in only one patient (in the A30 group) no inhibition was observed. The higher degree of TS inhibition in the in A120 group (as well as inhibition in all patients) is in line with the higher MeTHF polyglutamate levels, although the lower ratio of poly-/mono glutamated MeTHF might give another impression. Apparently, polyglutamate formation is saturated in the A120 group, or the higher polyglutamate level may enable folylpolyglutamate hydrolase (FPGH) to catalyze degradation faster. A similar pattern (saturation and higher degradation of polyglutamated FTHF)) was found for FTHF levels, where the highest level and the lowest poly-/monoglutamate ratio was found in the LV60 group

The present results indicate that a bolus Arfo 120 mg/m^2^ could be more effective than LV 60 mg/m^2^ in combination with bolus 5-FU. However, it would have been interesting to compare this group to a group given an equivalent dose of 240 mg/m^2^ LV as well as with Isovorin which only contains the natural form of FTHF. Previously it has been shown that an equimolar dose of Isovorin compared to the racemic mixture led to a comparable TS inhibition [27].

To increase the understanding of the impact of different folate forms on TS inhibition, analysis of deoxyuridine (a surrogate marker for TS inhibition), incorporation of 5FU into RNA (as a marker for systemic toxicity) and DNA (as a marker for DNA damage) as well as expression of genes associated with folate and 5-FU metabolism will be analyzed in samples deriving from the Modelle-001 Trial.

In conclusion, the Modelle-001 Trial demonstrated significantly higher levels of MeTHF (both mono- and polyglutamates) in metastases following Arfo compared to LV. This resulted in a greater increase TS inhibition in metastases although not statistically significant. All patients in the A30 group showed TS inhibition in metastases, whereas several patients in the LV60 group had no TS inhibition. The median TS inhibition was highest in the A120 group.

## Supplementary information

Supplementary Figures_Tables

## Data Availability

Clinical data have been collected from patients in accordance with agreements and regulations for consent that prohibit transfer to third parties. Other datasets used or analyzed during the study are available from the corresponding author upon reasonable request.
