# Supplementary material for: Increased potentiation of 5-fluorouracil induced thymidylate synthase inhibition by 5,10-methylenetetrahydrofolate (arfolitixorin) compared to leucovorin in patients with colorectal liver metastases; The Modelle-001 Trial
Source: BJC Rep. 2024 Nov 20;2:89. doi: 10.1038/s44276-024-00111-4 (PMC11579015; doi:10.1038/s44276-024-00111-4)
Supplement: Supplementary file 1 — Supplementary Figures_Tables [file 44276_2024_111_MOESM1_ESM.pdf]

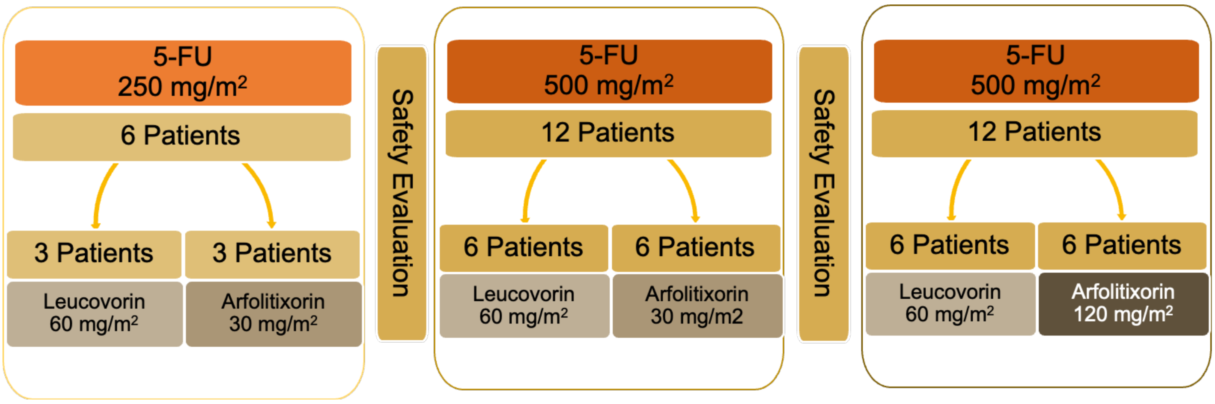

Supplementary Figure. 1. Flow chart of the study design.

Folate levels and ratio of poly-/monoglutamated folates in liver parenchyma

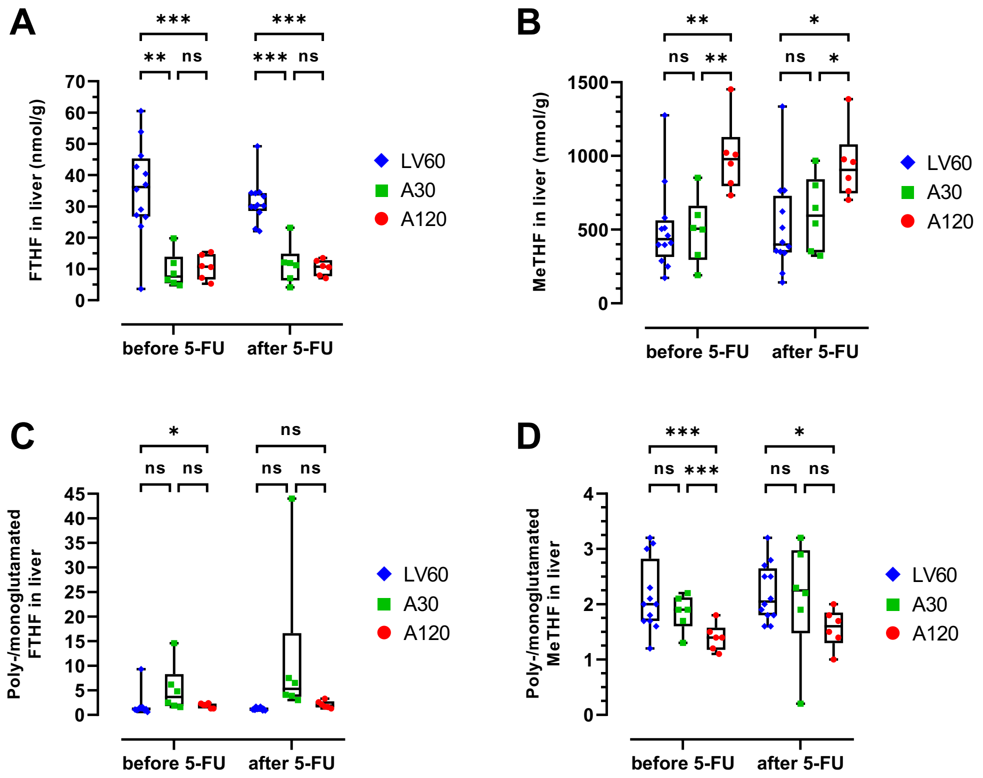

Supplementary Figure 2. Comparison of total (mono- and polyglutamates) 5-formyltetrahydrofolate (FTHF) and 5,10-methylenetetrahydrofolate (MeTHF) concentrations in liver parenchyma (**A,C**) and metastases (**B,D**) after intravenous bolus injections of 60 mg/m^2 LV (LV60), 30 mg/m^2 Arfo (A30), or 120 mg/m^2 Arfo (A120) followed by bolus injections of 500 mg/m^2 5-FU. Fig C,D shows the ratio of poly/monoglutamates. Data are presented as box-and-whisker plots with median, 25% and 75% quantiles (box), minimum and maximum (whisker), and outliers. Symbols represents individual values. Significant differences between respective groups are denoted by by asterisks; *p<0.05; **p<0.01; ***p<0.001.

Supplementary Table 1. Time until biopsy specimen collection after folate and 5-FU injection

		Time passed (minutes), median (range)		
Group	n	After folate	After 5-FU	After folate and 5-FU
LV60	12	75 (61-108)	109 (64-276)	195 (132-384)
A30	6	83 (60-168)	91 (60-108)	168 (142-240)
A120	6	61 (55-103)	91 (72-146)	156 (131-249)

Supplementary Table 2

TS inhibition in liver parenchyma after 5-FU injection

Group	n	TS inhibition (%)	p
		Median (range)	
LV60	11	45.5 (0-66.3)	0.80[a]
A30	6	49.0 (0-100)	0.20[b]
A120	6	51.2 (0-72.1)	0.94[c]

[a]LV60 vs A30, [b]A30 vs A120, [c]LV60 vs A120.
